# Spatial Imaging and Quantification of Hydrogen Peroxide in *Arabidopsis* Roots: From Sample Preparation to Image Analysis

**DOI:** 10.21769/BioProtoc.5659

**Published:** 2026-04-20

**Authors:** Mario Fenech, Vitor Amorim-Silva

**Affiliations:** Área de Mejora y Fisiología de Plantas, Instituto de Hortofruticultura Subtropical y Mediterránea “La Mayora”, Universidad de Málaga‐Consejo Superior de Investigaciones Científicas (IHSM La Mayora UMA‐CSIC), Universidad de Málaga, Campus Teatinos, 29010, Málaga, Spain

**Keywords:** Hydrogen peroxide, DAB staining, Image analysis, ROS quantification, FIJI/ImageJ

## Abstract

Reactive oxygen species (ROS) are central regulators of plant development and stress responses, with hydrogen peroxide (H_2_O_2_) acting as a key signaling molecule whose spatial distribution determines adaptive versus damaging outcomes. Accurate detection of H_2_O_2_ at tissue and cellular resolution is therefore essential for understanding redox-dependent regulation of plant growth. A variety of techniques have been used to monitor H_2_O_2_, including bulk spectrophotometric and fluorometric assays, genetically encoded sensors for real-time measurements, and chemical probes for in situ detection. While these approaches differ in sensitivity, specificity, and temporal resolution, many are limited by a lack of spatial information, technical complexity, or dependence on transgenic material. Here, we present a detailed protocol for 3,3′-diaminobenzidine (DAB)-based histochemical detection of H_2_O_2_ in seedling roots, covering staining, imaging, and semi-quantitative image analysis using open-source software (FIJI/ImageJ). The method relies on peroxidase-mediated oxidation of DAB, resulting in a stable, light-resistant, and insoluble precipitate that enables visualization of H_2_O_2_ accumulation with high spatial resolution. This protocol provides a robust, accessible, and genetically independent approach for spatial analysis of H_2_O_2_ in plant tissues. Its simplicity, compatibility with diverse genotypes and treatments, and suitability for semi-quantitative analysis make it a valuable tool for examining the spatial distribution of H_2_O_2_, thereby providing spatial insight into redox-related regulatory processes during plant development and stress responses.

Key features

• Built upon methods developed by Thordal-Christensen et al. [1] and Daudi and O’Brien [2], with a specific focus on root staining.

• Includes a downstream image analysis pipeline for semi-quantitative H_2_O_2_ measurement in DAB-stained roots using the open-source software FIJI/ImageJ.

• Provides detailed, step-by-step video tutorials for image analysis in FIJI/ImageJ.

• Includes a Fiji/ImageJ script (Macro 1) for automating the application of fixed-intensity scaling using Spectrum LUT.

## Graphical overview


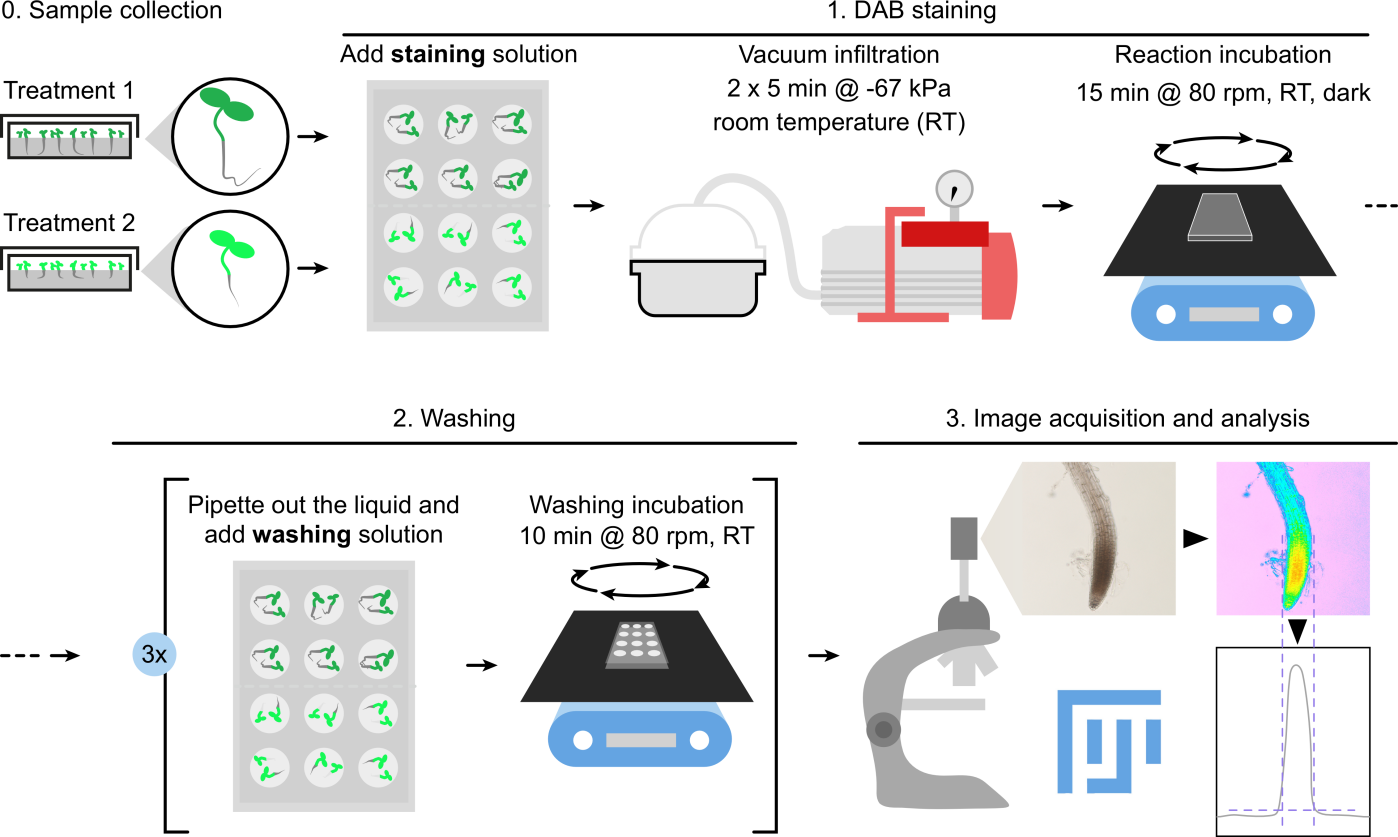


## Background

Reactive oxygen species (ROS) lie at the core of redox biology. In plants, ROS fulfill a dual role, acting both as signaling molecules and as cytotoxic agents [3]. Owing to its relatively higher stability compared to other ROS, hydrogen peroxide (H_2_O_2_) represents a central regulator of plant development and stress responses [4]. H_2_O_2_ can directly interact with proteins, thereby modifying their structure and downstream signaling pathways [3]. Consequently, the spatiotemporal regulation of H_2_O_2_ accumulation determines whether plant cells mount appropriate responses to environmental cues or instead experience oxidative damage leading to cell death. Accurate quantification of H_2_O_2_ with spatial resolution is therefore essential for understanding how ROS mediate cell-specific adjustments of plant growth under changing conditions.

A wide range of approaches has been developed to detect and quantify H_2_O_2_, each with distinct advantages and limitations. In general, substrate specificity and spatial resolution are the primary factors guiding method selection. Available techniques include spectrophotometric assays, enzyme-coupled fluorometric assays, imaging of genetically encoded sensors that allow real-time detection in living tissues, and histochemical staining methods that provide spatial information. Accordingly, the choice of assay depends on whether the experimental focus lies on bulk quantification, dynamic measurements, or spatial localization.

Classical spectrophotometric assays, such as the titanium–peroxide complex method [5], enable straightforward and cost-effective bulk quantification of H_2_O_2_ but lack spatial resolution. Enzyme-coupled fluorometric assays, including Amplex Red and Amplex Ultra Red [6–8], or enzyme-independent assays, like the europium–tetracycline complex [7], provide high sensitivity and accurate quantification at low H_2_O_2_ concentrations; however, they require tightly controlled reaction conditions and are generally unsuitable for in situ applications. Genetically encoded sensors, such as HyPer [9] and roGFP2-Orp1 [10], offer high H_2_O_2_ selectivity together with real-time, quantitative, and compartment-specific measurements in vivo [11]. Despite these advantages, their reliance on transgenic systems limits their applicability across diverse genetic backgrounds. Chemical probes, including H_2_DCF-DA (2′,7′-dichlorodihydrofluorescein diacetate), CM-H_2_DCF-DA (chloromethyl-H_2_DCF-DA), and DAB (3,3′-diaminobenzidine), permit intracellular ROS detection without genetic manipulation. H_2_DCF-DA and CM-H_2_DCF-DA are cell-permeable and widely used in living tissues [8,12–15]; however, their poor specificity and susceptibility to oxidation by multiple reactive species mean that they primarily reflect general redox status rather than H_2_O_2_ levels specifically.

Among these approaches, DAB staining has long been established as a classical method for detecting hydrogen peroxide in plant tissues [1,16]. The assay relies on a peroxidase-mediated reaction in which DAB is oxidized by H_2_O_2_, resulting in the formation of an insoluble brown precipitate. This enables spatial visualization of H_2_O_2_ accumulation in roots (e.g., [17,18]) as well as in leaves and other pigmented tissues following chlorophyll removal [2,19–21]. Although DAB staining is semi-quantitative and dependent on endogenous peroxidase activity, its simplicity, robustness, and capacity for spatial resolution have led to its widespread use in studies of plant oxidative stress [22].

Despite its widespread use, DAB staining protocols are often applied qualitatively or rely on simplified quantification approaches that lack standardized image analysis workflows, particularly for roots. Here, we present a refined and accessible protocol for semi-quantitative H_2_O_2_ detection in *Arabidopsis thaliana* roots that builds on established DAB staining methods while placing specific emphasis on root tissues and reproducible downstream analysis. Compared with bulk biochemical assays or genetically encoded sensors, this approach offers a favorable balance between spatial resolution, experimental accessibility, and applicability across diverse genetic backgrounds without the need for transgenic lines. Although DAB staining remains inherently dependent on endogenous peroxidase activity and does not provide absolute H_2_O_2_ concentrations, our protocol minimizes variability by integrating controlled staining conditions with a standardized, background-corrected image analysis pipeline implemented in open-source FIJI/ImageJ. This protocol provides a complete workflow, including DAB staining and washing steps for roots, detailed guidance on image acquisition, a transparent and reproducible quantification strategy (ROI definition, background subtraction, peak integration), and step-by-step video tutorials to facilitate adoption and reproducibility across laboratories.

## Materials and reagents

**Biological materials**

1. Previously grown 7-day-old *Arabidopsis thaliana* seedlings

*Note: We also successfully tested this protocol using 3–7-day-old seedlings. Moreover, older seedlings can be used when larger cell-culture wells are employed. For details on the growth conditions used to quantify H_2_O_2_ in this protocol, see [18].*

**Reagents**

1. HCl 37% (v/v) (Panreac AppliChem, catalog number: 471020.1611, CAS: 7647-01-0)

2. 3,3′-Diaminobenzidine (DAB) (Sigma-Aldrich, catalog number: D8001, CAS: 91-95-2)

*Note: DAB should be stored at room temperature (RT). The bottle must be kept tightly closed and stored in a dry, well-ventilated place, away from direct light and moisture, to prevent degradation. Other variants of the product exist, so it is important to note the exact reference. It is recommended to consult the Certificate of Analysis for the specific batch to verify the retest or expiry date.*

3. Ethanol (VWR ethanol AnalaR Normapur, catalog number: 20823.293)

4. Lactic acid (Panreac AppliChem, catalog number: 141034, CAS: 79-33-4)

5. Glycerol (Fisher BioReagents, catalog number: BP229-1, CAS: 56-81-5)

6. Deionized water (dH_2_O)

**Solutions**

1. Staining solution (see Recipes)

2. Washing solution (see Recipes)

3. Storage solution (see Recipes)

**Recipes**

**1. Staining solution**

**Table:** 

Reagent	Final concentration	Quantity or volume
DAB	1 mg/mL	20 mg
HCl (37%)	8 mM (adjust to reach pH 3.8)	16 μL
Total	n/a	20 mL

Dissolve DAB at RT with vigorous stirring and protect from light. Prepare fresh; storage at 4 °C for up to 2 days was tested without obvious loss of performance. Allow at least 2 h to ensure complete dissolution of DAB.

*Note: A freshly prepared staining solution should ideally be clear to very light brown. Do not use it if it darkens and turns reddish-brown, which usually indicates oxidation.*

**2. Washing solution**

**Table:** 

Reagent	Final concentration	Quantity or volume
Ethanol	60% (v/v)	30 mL
Lactic acid	20% (v/v)	10 mL
Glycerol	20% (v/v)	10 mL
Total	n/a	50 mL

The volumetric ratio (v/v/v) of ethanol:lactic acid:glycerol is 3:1:1. The solution can be stored at RT for long-term use (up to 6 months) in a sealed container.

**3. Storage solution**

**Table:** 

Reagent	Final concentration	Quantity or volume
Glycerol	60% (v/v)	30 mL
Total	n/a	50 mL

The solution can be stored at RT for long-term use (up to 2 years). After addition to samples, the mounted material can be stored at 4 °C for 7–10 days before fungal growth typically occurs.

**Laboratory supplies**

1. Cell culture multi-well plates (6- or 12-well plates to allow orbital movement) (BioFil, catalog numbers: TPC 011006 and TPC 011012)

2. 10 μL pipette tips (LLG, catalog number: 4668775)

3. 200 μL pipette tips (DasLab, catalog number: 162001)

4. 1 mL pipette tips (DasLab, catalog number: 162222)

5. Aluminum foil

6. 10 mL serological pipettes (LLG, catalog number: 6.268 240)

7. 50 mL serological pipettes (LLG, catalog number: 9.380 443)

8. (Optional) 50 mL conical plastic tubes (for storing solutions)

9. Glass slides and coverslips

10. Scalpel

## Equipment

1. Fume hood (Walder, model: scala)

2. Water deionizer/filter/Milli-Q system (Merck Millipore, model: Milli-Q^®^ IQ 7005)

3. Precision scale (Linear scale micrometer ruler 1 mm/0.01 mm) (WPI, catalog number: 500828)

4. 50 mL measuring cylinder (Normax, catalog number: 4.5149617)

5. 100 mL beaker (Normax, catalog number: 3.2110624N)

6. Microspoon or spatula (Carl Roth, catalog number: PX33.1)

7. Tweezers (to transfer seedlings into plates)

8. Pipette controller (Fisherbrand, catalog number: 15840053)

9. 1 mL micropipette (Gilson, model: PIPETMAN^TM^ P1000)

10. 200 μL micropipette (Gilson, model: PIPETMAN^TM^ P200)

11. 10 μL micropipette (Gilson, model: PIPETMAN^TM^ P10)

12. Magnetic stirrer and magnets [Merck, IKA^®^, catalog number: Z672378 and catalog number: Z744785 (Magnetic Stir Bar)]

13. Orbital shaker (VWR, model: 5000 Advanced Orbital Shaker)

14. 50/100 mL screw-cap glass bottles (for solution storage)

15. Timer

16. Vacuum pump (GAST, model: DCA-P504-BN)

17. Vacuum chamber (Merck, BRAND^®^ desiccator, catalog number: BR65810)

18. Microscope (10× objective, 0.63× ocular; transmitted light, not reflected) and microscope-compatible camera

*Note: We used a Zeiss Axio Scope A1 vertical microscope equipped with an AxioCam503 Color camera and Zeiss Zen Blue software.*

19. Computer

*Note: Image analyses described here were successfully performed without limitations on two different workstations: (1) ACER Nitro AN515-52, CPU: c2.30GHz Intel(R) Core(TM) i5-8300H; RAM: 32 GB 2666 MHz SO-DIMM DDR4; GPU: NVIDIA GeForce GTX 1050 (4096 MB GDDR5); OS: Windows 11 Pro (Version 10.0.26200); and (2) MacBook Pro, CPU: 1.4 GHz Quad-Core Intel Core i5; RAM: 16 GB 2133 MHz LPDDR3; GPU: Intel Iris Plus Graphics 645 (1536 MB); OS: macOS (Version 13.7.8).*

## Software and datasets

1. FIJI/ImageJ [23], open source ( https://imagej.net/software/fiji/ ). Bio-Format Importer and Colour Deconvolution H DAB vector are built-in plugins in FIJI/ImageJ v1.54p or later.

## Procedure

**A. Staining**

1. Prepare the staining solution at least 2 h before starting the experiment (see Recipes). In a 6- or 12-well plate, add 1 mL of staining solution to as many wells as needed.

**Caution:** DAB is suspected of causing genetic defects and cancer and must be handled using appropriate personal protective equipment (PPE).

*Notes:*

*1. The initial volume can be adjusted depending on well size and the number of seedlings. It is essential that the bottom of each well is fully covered with liquid so that roots do not contact the plastic surface, which may cause dehydration and damage.*

*2. While roots are flexible and can fit into small wells, long hypocotyls (e.g., seedlings germinated in darkness) are more prone to bending and mechanical damage. In such cases, consider using 6-well plates.*

**Figure 1.**
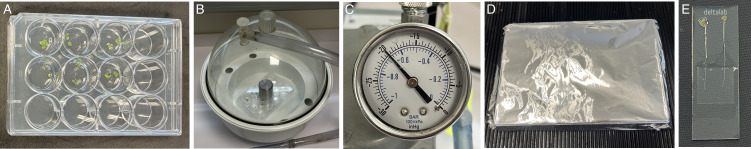
Experimental procedure for DAB staining in *Arabidopsis thaliana* seedlings using vacuum infiltration. (A) Seven-day-old seedlings subjected to control (top wells) or treatment (bottom wells) conditions are immersed in 1 mL of staining solution. Seedlings aged 3–7 days are commonly used in this protocol; however, this developmental stage is provided as an example and does not represent a strict requirement. (B) After closing the multi-well plate, it is placed inside a vacuum chamber, and a heavy metal weight is positioned on top to secure the lid upon vacuum release. Securing the lid is not strictly required, but it prevents liquid or sample spillage without significantly impairing vacuum efficiency and fast processing through steps. Alternatively, two opposite sides of the lid can be taped onto the plate in a non-airtight manner, allowing air to circulate in and out of the plate. Both methods work well; independently of the choice, we discourage sudden vacuum release prior to opening the chamber. (C) A manometer connected to the vacuum pump is used to ensure that infiltration reaches at least -67 kPa (equivalent to -500 mmHg or -20 inHg). (D) During staining incubation, plates are wrapped in aluminum foil and incubated in the dark at RT for 15 min at 80 rpm on an orbital shaker. (E) For microscope imaging, seedlings are mounted on glass slides on 20–50 μL drops of dH_2_O on a glass slide, spaced approximately 0.5 cm apart, and root tips are covered by a coverslip. Roots may be detached from the seedling or mounted intact for subsequent whole-seedling imaging. Single-plane images focused on the meristematic region of the root were acquired using a 10× objective and 0.63× ocular.

2. Using tweezers, gently transfer 3–5 seedlings into each well, minimizing damage. Keep experimental categories (e.g., genotypes, treatments, and time points) in separate wells, as the incubation step will mix all seedlings within a well (Figure 1A).

*Note: Seedling density can be adjusted depending on the experimental setup. Avoid overcrowding, as longer roots tend to intertwine and become difficult to separate without breakage after staining.*

3. If necessary, add additional staining solution to ensure that seedlings are fully immersed for homogeneous exposure.

4. To promote DAB uptake, vacuum-infiltrate the seedlings. Cover the plate with its lid and place it inside a vacuum chamber (Figure 1B). Turn on the vacuum pump and allow the pressure to reach -67 kPa (equivalent to -500 mmHg or -20 inHg) for 5 min (Figure 1C).

*Notes:*

*1. Keeping the plate covered prevents liquid or sample displacement upon vacuum release and does not interfere with vacuum formation inside the plate. To further protect against sudden air influx, place a heavy metal weight on top of the lid (Figure 1B).*

*2. If a manometer is not available, ensure that the chamber maintains a tight seal by gently lifting the chamber by its lid before starting the timing.*

**Caution:** Some vacuum pumps (e.g., oil-based pumps) require controlled pressure restoration prior to disconnection. Slowly allow air to re-enter the chamber before disconnecting the pump.

5. Gently swirl the plate and repeat step A4 for a total of two vacuum infiltration cycles.

6. Confirm that the staining solution volume is compatible with gentle orbital mixing: seedlings should move freely without liquid spilling (excess volume or speed) or sticking to the well walls (insufficient volume).

7. Wrap the plate completely in aluminum foil to ensure darkness and incubate at RT for 15 min at 80 rpm on an orbital shaker (Figure 1D).

*Note: This protocol used a VWR 5000 Advanced Orbital Shaker with a 25 mm orbit diameter. When using shakers with different orbit diameters, adjust the rpm to maintain gentle orbital mixing.*

**B. Washing**

1. Gently pipette out and discard the staining solution from each well, taking care not to aspirate the roots to avoid tissue damage.

*Note: After DAB oxidation, the brown precipitate is a stable and light-resistant end product; from this step onward, samples can be handled under normal light conditions.*

2. (Optional prewash) Add 1 mL of washing solution to each well and gently swirl the plate.

3. Carefully remove and discard the liquid, then add 1 mL of fresh washing solution to each well.

*Note: The washing volume can be adjusted according to the criteria described in step A6 to ensure compatibility with gentle orbital mixing.*

4. Incubate the plate at RT for 10 min at 80 rpm on an orbital shaker.

*Note: This protocol focuses on root tissues. In protocols designed for photosynthetic tissues, a boiling step at 95 °C for 15 min has been recommended to remove chlorophyll (see [2]).*

5. Repeat steps B3–4 for a total of three washing cycles.

6. Carefully replace the washing solution in each well with 1 mL of storage solution.

**Pause point:** At this stage, samples can be stored at 4 °C for up to one week, or longer if no fungal growth is observed.

**C. Root imaging**

1. Because of the difference in width between aerial and underground organs, mounting whole seedlings can cause the coverslip to tilt, resulting in increased depth of field and reduced in-focus root regions. Therefore, we recommend detaching roots just below the hypocotyl–root junction using tweezers. To do that, seedlings can be gently transferred to an agar plate, stretched, and cut using a scalpel. Alternatively, a small coverslip (e.g., 20 × 20 mm) can be used to cover only the root tips of intact seedlings (Figure 1E).

2. To mount the samples, place 20–50 µL drops of dH_2_O on a glass slide, spaced approximately 0.5 cm apart. If necessary, gently blot each root to remove excess glycerol and transfer roots individually into separate water drops. Carefully place a coverslip over the samples and, if needed, add additional dH_2_O to ensure proper mounting (Figure 1E).

*Note: We do not recommend mounting detached roots from different experimental categories on the same slide, as placement of the coverslip may rearrange and mix samples.*

3. Image roots using a conventional transmitted-light microscope (see Equipment section for details) that provides a sufficiently large field of view to capture the root apical meristem, elongation zone, and part of the differentiation zone. Adjust the field of view to the region of interest and acquire images using the brightfield (BF) channel.

*Note: The images used in the downstream analysis pipeline were single-plane images. However, users may choose to acquire Z-stack images if their goal is to construct 3D models of hydrogen peroxide accumulation in roots.*

**Critical:** Carefully adjust illumination intensity, gain, and exposure time to avoid signal saturation and apply these settings consistently across experimental categories. As in other quantitative or semi-quantitative imaging approaches, saturation severely compromises comparisons between experimental categories.

**D. Image analysis**

1. To analyze DAB staining intensity using FIJI (Video 1), images may first require preparation. Some microscopy formats are saved as 16-bit pseudo-RGB multichannel stacks, whereas Colour Deconvolution requires an RGB Color image type. Convert images as follows:

a. Microscopy formats (e.g., .czi, .lsm): Open the file using FIJI’s Bio-Formats Importer as a Hyperstack. In FIJI’s toolbar, select *File* > *Import* > *Bio-Formats* (or *Plugins* > *Bio-Formats* > *Bio-Formats Importer*), then double-click the file. In the *Bio-Formats Import Options* window, set *View stack with* to *Hyperstack* and *Color mode* to *Default*, uncheck all other options, and click *OK*. Once imported, select the image window and convert it to RGB by clicking *Image* > *Type* > *RGB Color*. A new RGB copy of the image will appear.

*Note: If direct conversion to RGB Color is not possible, first convert the image to 8-bit (*Image *>* Type *>* 8-bit*), then convert to RGB Color.*

b. Other image formats (e.g., JPG, TIF, PNG): Ensure that the image type is RGB Color; if not, convert it accordingly (*Image* > *Type* > *RGB Color*).

**Video 1.**
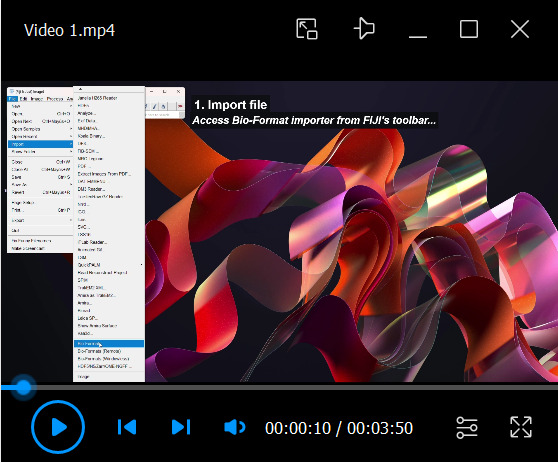
Estimation of DAB staining intensity in *Arabidopsis thaliana* roots using FIJI

2. Orient the root vertically to facilitate analysis. To do this, select *Image* > *Transform* > *Rotate*. In the pop-up window, enable *Preview* and adjust the rotation angle until the root is aligned vertically.

3. To separate DAB staining from other color components, apply Colour Deconvolution. Select *Image* > *Color* > *Colour Deconvolution*, then choose *H DAB* from the *Vectors* drop-down menu and click *OK*. This vector assumes dual staining with hematoxylin (H) and DAB and generates three 8-bit images: *Colour 1* (hematoxylin, blue–purple), *Colour 2* (DAB, brown), and *Colour 3* (residual, green). For subsequent analysis, retain two windows: the original RGB BF image [to define the region of interest (ROI)] and *Colour 2* (8-bit DAB) for quantification. Close all other windows.

4. Define the ROI on the BF image. Open the ROI Manager by selecting *Analyze* > *Tools* > *ROI Manager*. Using the rectangle selection tool, draw a rectangle wider than it is tall, spanning the root region to be analyzed, and click *Add* in the ROI Manager. An ROI ID will be assigned.

*Notes:*

*1. The ROI does not need to be defined on the BF image; however, doing so facilitates identification of specific cell types and root regions compared with the 8-bit DAB image. For example, in Video 1, the selected area encompasses the root apical meristem (RAM), whose boundary is defined by a duplication in epidermal cell length—features that are more readily identifiable in the BF window.*

*2. Due to the nature of this tool, the ROI must be a horizontal rectangle. As the rectangle approaches a square shape, the tool may fail to extract pixel values correctly.*

5. Select the *Colour 2* (DAB) image and click the corresponding ROI ID in the ROI Manager. The ROI defined on the BF image will be transferred to the DAB image. To quantify DAB staining intensity, select *Analyze* > *Gels* > *Select First Lane*; a “1” will appear at the center of the ROI. Then, select *Analyze* > *Gels* > *Plot Lanes*. The resulting plot represents the accumulated DAB staining intensity along the ROI.

*Note: Vertically aligned samples typically produce a narrow, leptokurtic peak, whereas slightly tilted samples generate broader, skewed peaks.*

**Critical:** Verify that the peak is not truncated at the top. Peak truncation indicates signal saturation during image acquisition, which will lead to underestimation of differences between experimental categories.

6. To subtract the background signal, use the *Straight Line* tool to draw a baseline at the base of the peak. Then, select the *Wand* tool and click inside the peak area. A dialog box will display the enclosed area, representing integrated DAB staining intensity. Copy this value into a spreadsheet for subsequent statistical analysis.

7. (Optional) Visual representation of DAB staining intensity (Video 2): In addition to quantitative measurements, a visual representation of relative DAB staining intensity can aid spatial interpretation of staining patterns within and beyond the ROI. This visualization uses the Spectrum look-up table (LUT) in FIJI and is intended for qualitative comparison only. A Fiji/ImageJ script for automating the application of fixed-intensity scaling using Spectrum LUT is available in the supplementary information section (Macro 1). To visualize DAB intensity using the Spectrum LUT without using Macro 1, proceed as follows:

**Video 2.**
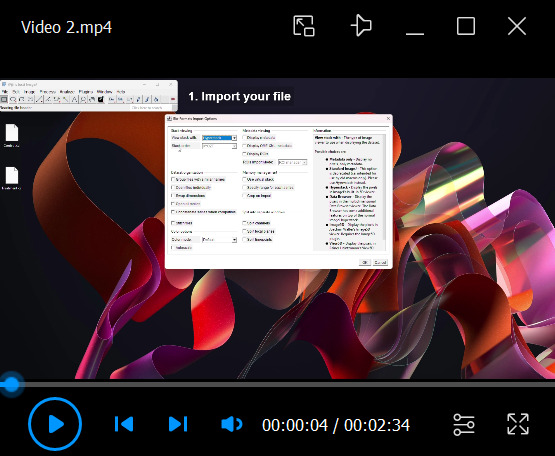
Application of Spectrum LUT in FIJI/ImageJ for visual representation of DAB staining intensity

a. Import the image as described in step D1 and convert it to 8-bit (*Image* > *Type* > *8-bit*).

b. Apply the Spectrum LUT by selecting *Image* > *Lookup Tables* > *Spectrum*.

*Note: In this LUT, red indicates high-intensity pixels and blue indicates low-intensity pixels. Images with saturated DAB signal (see Critical note in step D5) will display red regions corresponding to pixel saturation.*

c. To enhance visual differences between experimental categories, adjust brightness and contrast while ensuring consistent display settings across images. Open *Image* > *Adjust* > *Brightness/Contrast*, click *Set*, and define identical minimum and maximum display values for all images. If multiple images are open, selecting *Propagate to all other open images* applies the same settings uniformly. Otherwise, record the values and apply them manually. This ensures visual comparability without altering the underlying image data.

d. Save the image by selecting *File* > *Save As* > *JPEG* (or another desired format).

## Data analysis

Due to the intrinsic variability of DAB staining, some intensity measurements may represent technical anomalies. Removing such values can negatively affect the statistical power of the experiment; therefore, DAB intensity was measured in more than five biological replicates, and all observed patterns were confirmed in at least one independent experimental repetition. To objectively identify and remove values considered outliers that could introduce noise, datasets were screened using Tukey’s fences, a robust, distribution-free method based on quartiles. Briefly, outliers were defined as values below Q1 - 1.5 × IQR or above Q3 + 1.5 × IQR, where IQR is the interquartile range.

After removal of outliers, appropriate hypothesis-testing analyses were performed. Given that DAB intensity is a continuous variable, parametric tests (Student’s *t*-test or one-way ANOVA) were used to compare experimental groups when assumptions were met. Prior to analysis, data were assessed for homoscedasticity (α = 0.05) and statistical power (1 - β > 0.8, where β denotes the probability of accepting a false null hypothesis). When these criteria were satisfied, differences were analyzed using *t*-tests or ANOVA followed by Tukey’s post hoc test (α = 0.05).

Datasets that did not meet parametric assumptions were log_10_-transformed and reassessed. If transformed data still failed to satisfy homoscedasticity or power requirements, nonparametric tests were applied, including the Mann–Whitney–Wilcoxon test [24,25] or the Kruskal–Wallis test [26], followed by Dunn’s multiple-comparison test [27,28] at α = 0.05. For factorial designs requiring nonparametric two-way ANOVA, analyses were conducted using the Aligned Rank Transform approach (ARTool [29]).

All these analyses were performed using open-source software R Studio [30] using an in-house ad-hoc script.

## Validation of protocol

This protocol has been used and validated in the following research article:

• Fenech et al. [18]. *Arabidopsis* lines with modified ascorbate concentrations reveal a link between ascorbate and auxin biosynthesis. *Plant Physiology* (Supplementary Figure S12, panel A).

## General notes and troubleshooting

**General notes**

1. A 15-min incubation corresponds to the recommended and optimized duration for ROS detection in the root meristematic zone, preventing signal saturation. However, longer incubation times (e.g., up to 2 h) may be tested when analyzing plant tissues that accumulate low levels of H_2_O_2_. The protocol is also compatible with incubation at 4 °C, in which case incubation times should be adjusted accordingly.

2. When performing the protocol for the first time, we recommend including well-characterized controls, such as treatments that induce H_2_O_2_ accumulation (e.g., flg22) or genotypes with markedly reduced or exacerbated H_2_O_2_ levels (e.g., [31]).

3. FIJI keyboard shortcuts are not included in this protocol to maintain conciseness; however, users are strongly encouraged to use them to accelerate the image analysis workflow.

**Troubleshooting**

**Problem 1:** The DAB solution appears brown.

Possible causes: Old DAB stock or contaminated container.

Solutions: As best practice, prepare the DAB staining solution fresh. Minimize exposure to light and other oxidative agents during preparation. Thoroughly clean the container used for solution preparation and rinse it with deionized water before use. If the problem persists, verify the age and storage conditions of the DAB powder.

**Problem 2:** DAB does not dissolve.

Possible cause: Incorrect pH.

Solution: Measure the pH of the solution after adding HCl and before adding DAB. If the pH differs from 3.8, adjust it accordingly prior to adding DAB.

**Problem 3:** Weak or no DAB staining in roots.

Possible causes: Insufficient vacuum infiltration, low endogenous peroxidase activity, or staining solution that is too old or partially oxidized.

Solution: Ensure that vacuum infiltration reaches at least -67 kPa and that two infiltration cycles are performed. Confirm that the staining solution is freshly prepared and pale in color. If necessary, moderately increase the incubation time while carefully monitoring for signal saturation.

**Problem 4:** Excessive or saturated DAB staining.

Possible causes: Over-incubation in staining solution, high H_2_O_2_ accumulation due to stress during handling, or overexposure during image acquisition.

Solution: Reduce staining incubation time and handle seedlings gently to avoid mechanical or dehydration stress. During image acquisition, reduce illumination intensity and exposure time to avoid signal saturation (see Critical note in step C3).

**Problem 5:** Uneven staining between seedlings within the same well.

Possible causes: Incomplete immersion of seedlings, insufficient orbital mixing, or differences in developmental stage due to uneven germination.

Solutions: Ensure seedlings are fully immersed in the staining solution and that the liquid volume allows gentle orbital movement without spillage. Make sure seedlings are not sticking to the well walls, reduce seedling density per well if necessary, and gently separate seedlings before incubation. Use high-quality seed batches to promote uniform germination.

**Problem 6:** Tissue damage or broken roots after staining.

Possible causes: Excessive vacuum strength or abrupt pressure release, overcrowding, or aggressive handling with tweezers.

Solution: Apply vacuum gradually and restore pressure slowly. Reduce the number of seedlings per well, handle roots gently during transfers, and avoid aspirating roots when pipetting out solutions. Consider using larger wells for longer seedlings.

**Problem 7:** Roots move during mounting, complicating imaging.

Possible causes: Excess glycerol remaining on the tissue or excessive liquid under the coverslip.

Solution: Gently pad-dry roots before mounting to remove excess glycerol. Use minimal dH_2_O for mounting and carefully lower the coverslip to avoid sample displacement.

**Problem 8:** *Colour Deconvolution* does not work.

Possible cause: The image is not in RGB color format.

Solution: Convert the image to RGB Color using FIJI, as described in step D1.

**Problem 9:** FIJI displays a noisy curve rather than a sharp peak when plotting DAB intensity.

Possible cause: The ROI is too close to a square rather than an elongated horizontal rectangle.

Solution: When possible, extend the length of the ROI beyond the sample to ensure a clearly horizontal rectangular shape.

**Problem 10:** Error message appears when clicking *Plot Lanes*, stating that “Select First Lane” must be used first.

Possible cause: The “Select First Lane” command was not reapplied after switching to a new image. This command must be executed once per image, even when using the same ROI.

Solution: Use the “Select First Lane” command each time you switch to a different image.

**Problem 11:** The DAB intensity curve appears as a valley instead of a peak.

Possible cause: FIJI is quantifying bright (white) pixels rather than DAB-positive (brown) pixels.

Solution: In FIJI, go to *Analyze* > *Gels* > *Gel Analyzer Options*… and enable the option “Invert peaks.”

**Problem 12:** Peak area measurements do not accumulate in the Results window.

Possible cause: In Fiji/ImageJ, the *Gel Analyzer* tool operates in a session-based manner. In this protocol, each image produces a single peak and therefore requires initialization of a new gel session (*Analyze* → *Gels* → *Select First Lane*). When a new session is started, the Results window may overwrite previously recorded peak areas. Consequently, when integrating peaks from different images (i.e., different sessions), only the most recently measured value may be displayed.

Solution: Because each image requires an independent gel session, peak areas cannot be reliably accumulated automatically across multiple plot windows. We therefore recommend recording each peak area sequentially in an external spreadsheet during analysis. Alternatively, as a practical workaround, measurements can be forced to accumulate within the same Results window by sequentially activating each plot window before repeating the measurement. In practice, the first peak measured in a newly opened plot window overwrites the existing entry in the Results table. However, once a plot window has been activated and measured once, subsequent measurements from previously activated windows will append to the Results table rather than overwrite it. Users should therefore verify that values are accumulating correctly before exporting the dataset as a .csv file.

## Supplementary information

The following supporting information can be downloaded here:

1. Macro 1. FIJI/ImageJ script for automated application of fixed-intensity scaling using Spectrum LUT
